# Advancing mRNA vaccines for infectious diseases: key components, innovations, and clinical progress

**DOI:** 10.1042/EBC20253009

**Published:** 2025-05-01

**Authors:** Sha Li, Lu Zheng, Jingyi Zhong, Xihui Gao

**Affiliations:** 1 Shanghai Pudong Hospital, Key Laboratory of Medical Molecular Virology (MOE/NHC/CAMS), School of Basic Medical Sciences, Shanghai Medical College, Fudan University, Shanghai, China; 2 Shanghai Institute of Infectious Disease and Biosecurity, Fudan University, Shanghai, China

**Keywords:** clinical trials, infectious diseases, mRNA, nanoparticles, vaccines

## Abstract

Vaccination remains a cornerstone in preventing infectious diseases and managing outbreaks. The COVID-19 pandemic has underscored the revolutionary impact of mRNA vaccine technology, which utilizes pathogenderived genomic sequences to generate specific antigens. This process involves *in vitro* transcription of mRNA, encoding target antigens that are subsequently encapsulated within lipid nanoparticles (LNPs) for efficient delivery into host cells. Once internalized, the mRNA enables antigen expression, triggering a robust immune response. This platform dramatically accelerates vaccine development timelines and offers unparalleled adaptability, making mRNA vaccines particularly advantageous in addressing emerging infectious diseases. The clinical success of BNT162b2 (Pfizer-BioNTech) and mRNA-1273 (Moderna) has fueled broader applications, including influenza, respiratory syncytial virus (RSV), Zika, and HIV. Notably, mRNA-1345 became the first FDA-approved RSV mRNA vaccine, while self-amplifying RNA and multivalent vaccines are advancing in trials. However, CureVac’s CVnCoV failed due to lack of nucleoside modifications, and mRNA-1325 (Zika) showed poor immunogenicity. Additionally, mRNA-1365 (RSV) faced an FDA clinical hold due to safety concerns. These cases highlight the need for continued optimization in sequence design, delivery, and safety assessment. Despite advancements, a key hurdle persists, including mRNA instability, ultra-low storage requirements, and LNP liver accumulation. Innovations such as lyophilization and selective organ targeting technology are being explored to improve stability extrahepatic delivery. This review examines mRNA vaccine optimization strategies, clinical progress, and challenges, providing insights into future developments in this evolving field.

## Introduction

Vaccination remains one of humanity’s most powerful tools in the fight against infectious diseases. According to the World Health Organization, vaccines prevent an estimated million of deaths annually [1]. However, traditional vaccine development encounters several challenges, including lengthy production timelines and the rapid mutation of viruses, particularly RNA viruses, which can render vaccines ineffective against emerging strains [2]. Additionally, safety concerns such as the risk of ‘virulence reversion’, where live attenuated vaccines might mutate back to a pathogenic form, further complicate conventional vaccine development (Table 1) [3].

**Table 1:** Comparison of prophylactic vaccine types for infectious diseases.

Vaccine type	Immune response	Stability	Safety	Examples
Live attenuated vaccines	Robust humoral and cellular immunity	Requires cold chain; moderate stability	Risk of reversion to virulence; not for immunocompromised individuals	Yellow fever, oral polio
Inactivated vaccines	Primarily humoral immunity	Stable; requires cold chain	No reversion risk; lower immunogenicity	Hepatitis A; rabies
Subunit vaccines	Primarily humoral immunity	High stable; no cold chain needed	Very safe; requires adjuvants	Hepatitis B; HPV; pertussis
Viral vector vaccines	Robust humoral and cellular immunity	Requires cold chain; moderate stability	Pre-existing immunity may reduce efficacy	COVID-19
LNP-mRNA vaccines	Robust humoral and cellular immunity	Requires ultra-low temperature storage; improving with lyophilization	Possible reactogenicity; concerns over LNP and PEG sensitivity	COVID-19

In recent decades, mRNA technology has emerged as a ground-breaking platform for vaccine and therapeutic development. Since the discovery of mRNA in 1961, its potential has been progressively explored, with early demonstrations of *in vitro* transcription (IVT) and successful validation in mice occurring in 1990s [4,5]. However, the clinical application of mRNA faced substantial hurdles, including poor cellular uptake due to its negatively charged phosphate backbone, susceptibility to enzymatic degradation, and rapid systemic clearance [6,7]. These challenges underscored the crucial need for an efficient and safe delivery system to fully unlock mRNA’s therapeutic potential.

The development of lipid-based delivery systems has been a major milestone in overcoming these obstacles. Liposomes were first explored for nucleic acid delivery in the 1970s, but their short half-life, high sensitivity, and a complex preparation process limited their practical use [8–10]. A significant breakthrough came in the 2010s with the development of four-component lipid nanoparticles (LNPs), which revolutionized nucleic acid delivery [11]. By 2018, the FDA had approved the first LNP-based siRNA therapeutic, paving the way for further advancements. The emergence of the SARS-CoV-2 pandemic in 2020 accelerated research into LNP-mRNA vaccines [12]. Compared with traditional vaccines, mRNA vaccines offer substantial advantages, such as bypassing the need for cell culture and enabling rapid large-scale production, dramatically reducing development timelines.

Despite these advantages, several challenges remain. Issues such as ultra-low temperature storage requirements, potential allergic reactions to LNP components, and the rapid waning of neutralizing antibody titers are the highlight areas that need further improvement [13–16]. Additionally, expanding mRNA vaccine distribution to extrahepatic tissues is another key frontier.

This review aims to provide a comprehensive analysis of the key components of LNP-mRNA vaccines, including antigen-encoding RNA design, LNP composition, and strategies for optimization. Furthermore, it will explore the current landscape of clinical trials for LNP-mRNA vaccines targeting infectious diseases, lessons from failed trails, and advances in extrahepatic tissues delivery, contributing to the ongoing advancements in this rapidly evolving field.

## Immune responses induced by LNP-mRNA vaccines

LNP-mRNA vaccines possess the remarkable ability to activate both the innate and the adaptive immune systems. Upon administration, exogenous mRNA that successfully enters cells is rapidly recognized by various pattern recognition receptors (PRRs), triggering a cascade of innate immune responses. Among these PRRs, Toll-like receptors 7 and 8 (TLR7/8) play a crucial role in detecting single-stranded RNA [17]. Additionally, double-stranded mRNA fragments can be sensed by TLR3 in the endosome, as well as cytoplasmic receptors such as retinoic acid-inducible gene I (RIG-I) and melanoma differentiation-associated gene 5 (MDA5). Activation of these diverse sensory pathways converges on shared downstream signaling nodes, culminating in the coordinated biosynthesis of type I interferons (primarily IFN-α/β) and pro-inflammatory cytokines. This inflammatory milieu functions as an ‘adjuvant-like’ environment, priming the immune system for subsequent adaptive responses.

Simultaneously, mRNA vaccines are internalized by local antigen-presenting cells (APCs), where they are translated into antigenic proteins. These proteins are then degraded into smaller peptides by the proteasome and presented on major histocompatibility complex class I (MHC-I) molecules to activate CD8^+^ cytotoxic T lymphocytes. In parallel, the antigenic peptides that enter the endosomal/lysosomal pathway are loaded onto MHC-II molecules for presentation to CD4^+^ helper T cells [18,19]. The expression of cytokines, such as type I interferons and co-stimulatory molecules, is crucial for APC-mediated T cell activation. Moreover, secreted antigenic proteins can bind to B cell receptors, triggering humoral immunity. CD4^+^ follicular helper T (Tfh) cells further assist B cells in producing high-affinity antibodies [20]. Research in animal models has demonstrated that LNPs induce high levels of IL-6 and other cytokines, which promote Tfh cells differentiation and germinal B cells formation, thereby accelerating the affinity maturation of antibodies. Moreover, type I interferon signaling enhances CD8^+^ T cells activation and proliferation, further strengthening immune protection [17,21].

**Figure 1:**
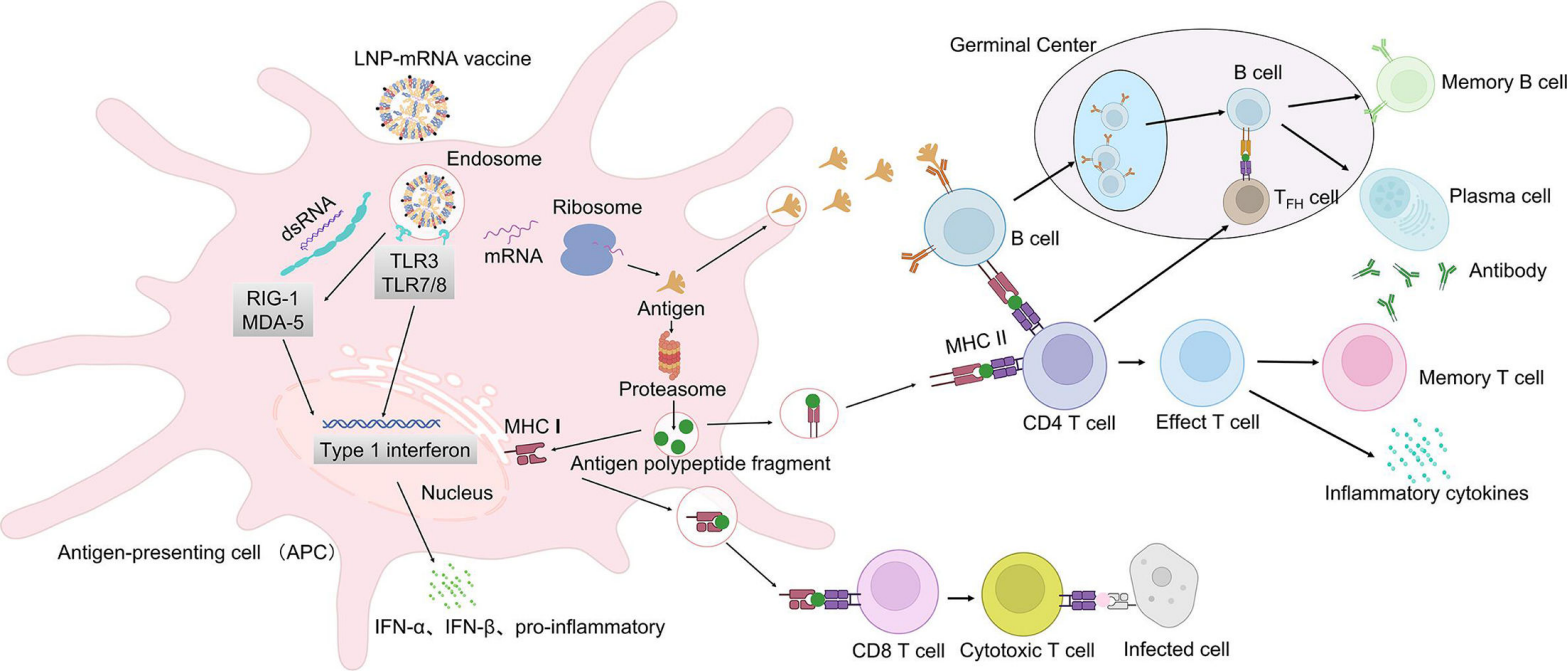
Immune responses induced by LNP-mRNA vaccines. Upon administration, LNPs facilitate mRNA uptake by antigen-presenting cells (APCs), leading to intracellular antigen expression. The mRNA or its byproducts can be recognized by pattern recognition receptors (PRRs) such as Toll-like receptors 7 and 8 (TLR7/8), retinoic acid-inducible gene I (RIG-I), and melanoma differentiation-associated protein 5 (MDA5), triggering an innate immune response characterized by the release of interferons (IFN-α/β) and pro-inflammatory cytokines. These signals enhance APC maturation, leading to the activation of CD4^+^ helper T cells (via MHC II presentation) and CD8^+^ cytotoxic T cells (via MHC I presentation), which collectively contribute to adaptive immune responses. Additionally, secreted antigenic proteins can activate B cells, leading to antibody production. This figure was created using BioRender.com.

In conclusion, mRNA vaccines effectively drive APCs maturation and lymphocytes activation. The innate immune system establishes an inflammatory environment rich in cytokines and interferons, providing a foundation for robust B and T cell responses. The coordinated activation of innate and adaptive immunity ultimately confers strong and durable protective immunity (Figure 1).

## Classification of LNP-mRNA vaccines

**Figure 2:**
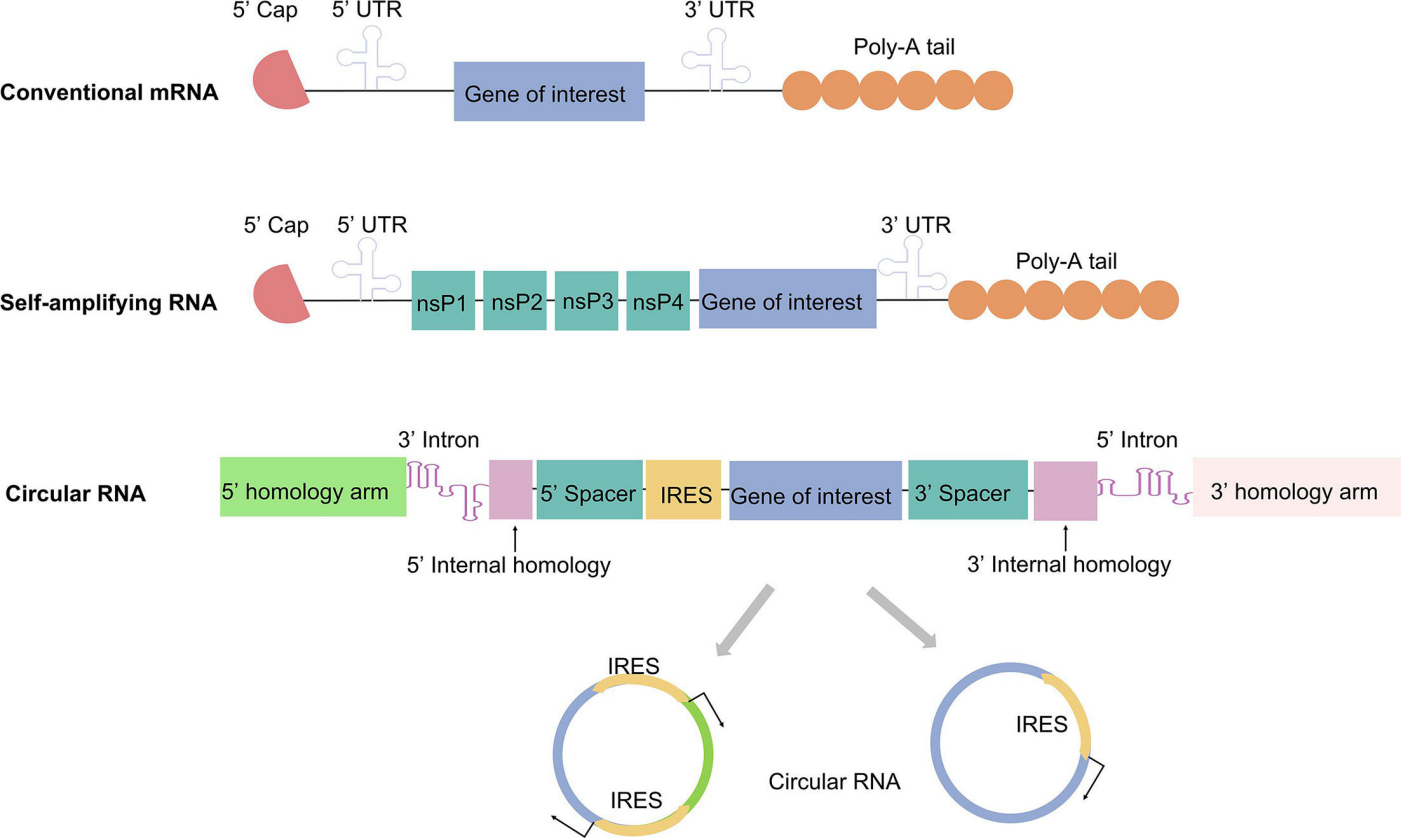
Types of RNA synthesized through *in vitro* transcription.

LNP-mRNA vaccines can be categorized based on RNA type. Currently, three major forms of RNA can be produced via IVT: conventional linear mRNA, self-amplifying RNA (saRNA), and circular RNA (circRNA) (Figure 2) [22].

**Conventional mRNA**, the most widely used type, consists of a 5´ cap, a 5´ untranslated region (UTR), an open reading frame (ORF) encoding the antigen, a 3´ UTR, and a poly (A) tail. While IVT technology enables rapid intracellular protein expression, conventional mRNA is highly susceptible to enzymatic degradation and requires high doses to achieve protective efficacy.

**saRNA**, derived from positive-sense RNA viruses, represents the next generation of RNA technology. By encoding a replicase while replacing viral structural genes with antigen sequences, saRNA can self-replicate within cells, significantly reducing the required vaccine dose and administration frequency [23]. This mitigates challenges such as the ‘PEG dilemma’ observed in repeated vaccinations [24]. Researchers have indicated that saRNA vaccines elicit robust immune responses at much lower doses than conventional mRNA vaccines. For instance, McKay et al*.* developed an saRNA-based SARS-CoV-2 vaccine that induced strong antibody titers, a Th1-biased immune response, and superior neutralization against pseudoviruses and wildtype strains, exceeding the antibody potency found in convalescent COVID-19 patients [25].

Additionally, saRNA generates immunostimulatory signals via dsRNA intermediates formed during replication, further enhancing the innate immune response [26–28]. Nevertheless, saRNA molecules are relatively large, typically exceeding 9 kb as they encompass both replicase and antigen sequences. This characteristic imposes higher performance requirements on delivery vectors. Their production process is more complex than conventional mRNA. Moreover, while saRNA-induced innate immunity can enhance immunogenicity, excessive activation may suppress antigen expression, requiring careful optimization [27].

**circRNA**, characterized by a closed-loop structure lacking a 5´ cap and 3´ poly (A) tail, exhibits enhanced nuclease resistance and prolonged *in vivo* stability [29]. Notably, circRNA vaccines remain at 4℃ for at least four weeks, whereas conventional linear mRNA-LNP vaccines degrade under similar conditions within three weeks [30]. Due to their closed structure, circRNAs evade innate immune recognition more effectively, reducing excessive inflammatory responses and potentially improving safety [31]. Besides, studies have demonstrated that engineered and optimized circRNA exhibits remarkable superiority in protein production. *In vitro*, the level of active protein generated by circRNA is substantially higher than that produced by an equivalent amount of linear mRNA. This advantage is observed not only *in vitro* but also *in vivo*, where circRNA shows both enhanced translational efficiency and prolonged protein expression [32].

Advancements in delivery systems have further expanded the potential of circRNA vaccines. For instance, a mannose-modified LNP vector has been developed to enhance lymph node targeting, leading to prolonged antigen availability and stronger Tfh and germinal center B cell responses. Mouse studies revealed that lyophilized mLNP-circRNA vaccines retained stability at 4℃ for up to 24 weeks without loss of immunogenicity [33]. These findings suggest that by enhancing delivery methods, such as targeted delivery and improved stability during lyophilization, circRNA vaccines can offer greater convenience for storage and transportation while preserving high efficacy. The concept of circRNA vaccines has been validated in multiple disease models. For example, Wei *et al.* developed a circRNA vaccine encoding the receptor-binding domain (RBD) of the SARS-CoV-2 spike protein. In studies with macaque monkey experiments, this vaccine successfully elicited high-titer neutralizing antibodies and a significant T cell response, and it offered effective protection against both Delta- and Omicron-mutant strains [30]. Despite these promising advances, circRNA vaccines remain in the early stages of research, with key challenges such as large-scale production, *in vivo* metabolism, and long-term safety requiring further investigation [34].

## Optimization of RNA

Enhancing the stability, reducing immunogenicity, and improving protein expression of mRNA can be achieved through various optimization strategies. Key approaches include nucleoside modification, codon optimization, and structural refinement [35–37].

### Nucleoside modification

A major challenge of exogenous mRNA is its innate immunogenicity, which can trigger excessive inflammatory responses and interfere with antigen expression, potentially reducing vaccine efficacy and causing adverse effects [38]. Researchers have indicated that inappropriate or excessive activation of type I interferons, such as IFN-α, may disrupt antigen expression, reduce vaccine effectiveness, and potentially lead to severe adverse effects [39]. To mitigate this, various chemical modifications have been introduced to reduce immunogenicity while maintaining translation efficiency [40]. For instance, natural adenosine can be replaced with N^1^-methyladenosine (m^1^A) or N6-methyladenosine (m^6^A), and cytosine can be substituted with 5-methylcytosine (5-mC) [41,42]. Additionally, uridine can be replaced with 5-methoxyuridine (5moU), pseudouridine (Ψ), or N^1^-methylpseudouridine (m^1^Ψ) [43]. Notably, both 5-mC and Ψ have been shown to reduce mRNA immunogenicity and enhance protein translation in both *in vivo* and *in vitro* studies [44].

### Optimization of codon

Codon usage can be tailored to host cell preferences to enhance translation efficiency and protein yield. Strategies include substituting rare codons with more prevalent ones (to prevent ribosomal stalling) and increasing GC content to improve stability. For instance, codon-optimized E7 genes have shown enhanced immunotherapeutic efficacy in HPV-16 vaccines [45]. COVID-19 . After purifiedmRNA vaccines developed by Moderna and Pfizer/BioNTech, codon optimization improved antigen expression and vaccine effectiveness [46]. Additionally, optimizing codon sequences can reduce unwanted activation by minimizing RIG-I-mediated innate immune activation [47].

### Optimization of non-coding regions

The non-coding regions of mRNA, including the 5´ cap, 5´ UTR, 3´ UTR, and 3´ Poly (A) tail, play essential roles in regulating mRNA stability, translation, and cellular function.

**5´ cap** particularly the m7G cap structure (cap-0, cap-1, and cap-2) protects mRNA from exonuclease degradation and enhances translation efficiency while reducing immunogenicity [48].

**UTRs** influence translation efficiency, stability and localization. Using highly expressed natural UTRs, such as α- and β-globin, can prevent degradation and improve mRNA longevity [49].

**Poly (A) tail,** its length typically around 100 nt, is crucial for mRNA stability and expression efficiency. However, studies suggest that a 75 nt poly (A) tail may achieve optimal expression in human cells, indicating a context-dependent balance [50,51]. Novel designs, such as segmented poly (A) tail, further extend stability and translation efficiency [52].

### Artificial intelligence-assisted mRNA optimization

Machine learning and deep learning algorithms are revolutionizing mRNA vaccine design by enabling precise sequence optimization.

Advanced models such as convolutional neural networks, residual neural networks, and graph neural networks can analyze large-scale sequence data to extract intricate features, including codon usage preferences and secondary structures. Leveraging these deep learning architectures, researchers can subsequently optimize mRNA stability and translation efficiency by fine-tuning its design based on the complex patterns identified [53]. For example, during COVID-19 vaccine development, researchers introduced the LinearDesign algorithm, which optimized mRNA stability and codon usage within 11 minutes, leading to a 128-fold increase in antibody titers in mice [54]. Additionally, machine learning models have been employed to fine-tune UTR design and stabilize ORF structures (*CDSFold* model) to enhance vaccine efficacy [55]. Epitope prediction is a cornerstone of mRNA vaccine design. As the specific regions on antigen proteins where antibodies bind, epitopes play a critical role in vaccine efficacy, making their precise identification essential. Commonly used prediction tools, such as DeepAb, BepiPred, and DiscoTope, employ deep learning algorithms to analyze complex datasets [56]. By integrating computational techniques with bioinformatics, these tools efficiently screen for candidate sequences with robust immunogenic potential, thereby significantly enhancing both the precision and efficiency of mRNA vaccine development.

## Formulation of LNPs

**Figure 3:**
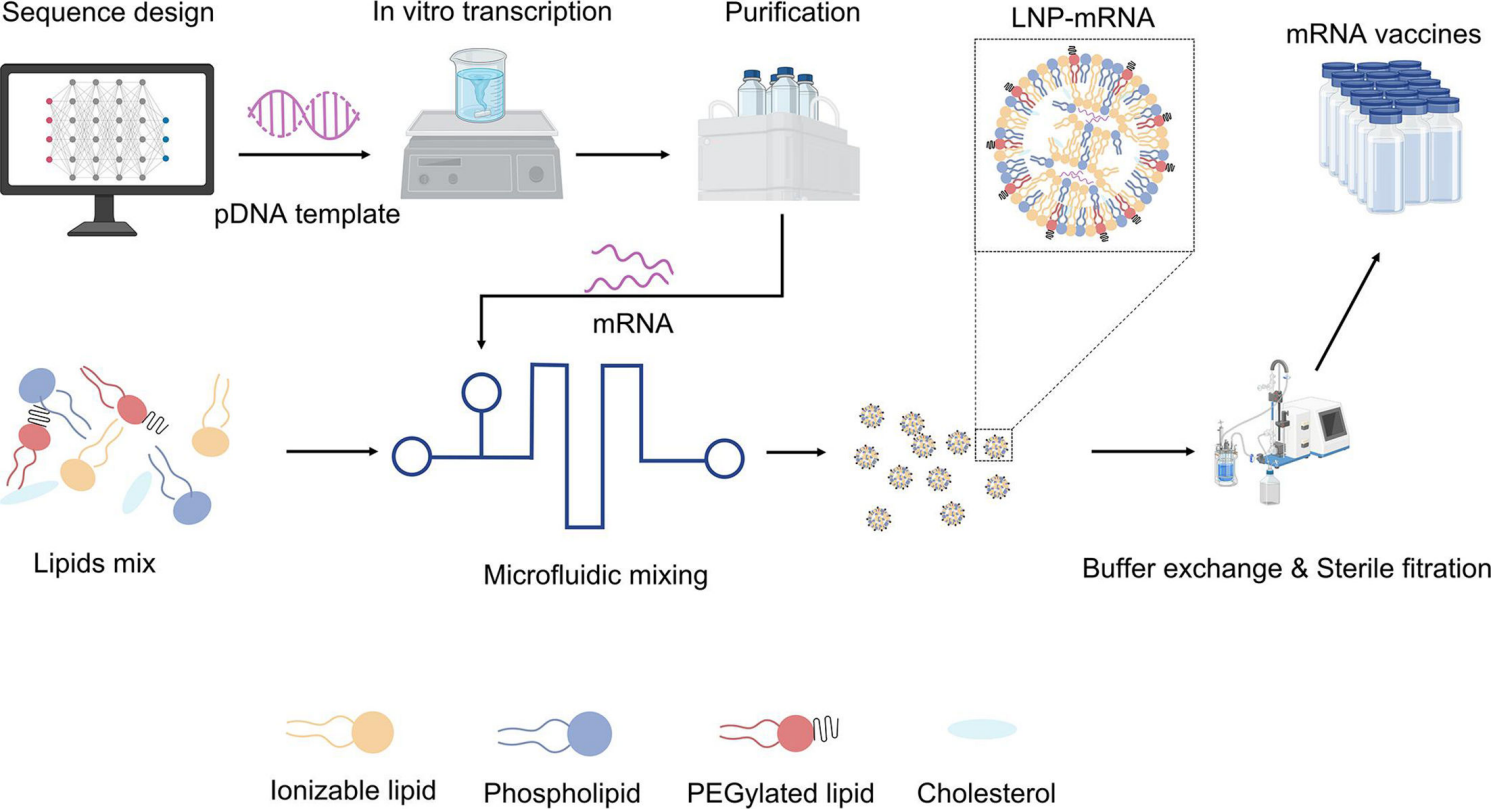
Preparation of LNP-mRNA vaccines. The DNA transcription template is designed based on the target antigen sequence, and then undergoes *in vitro* transcription (IVT) to generate mRNA. After purified, mRNA is subsequently mixed with lipid components to form LNP-mRNA complexes. Following LNP encapsulation, the formulation undergoes buffer exchange and sterile filtration to ensure safety and purity before being filled into vials for clinical use and distribution. This figure was created using BioRender.com.

LNPs are the most widely used delivery systems for mRNA vaccines, demonstrating efficient mRNA protection and intracellular delivery during the COVID-19 pandemic (Figure 3). Typically composed of ionizable lipids, cholesterol, helper lipids, and PEGylated lipids, LNPs enable rapid, scalable production with high biosafety. However, challenges such as hepatic accumulation, potential immunogenicity, and targeted delivery limitations remain. Addressing these issues is crucial for expanding LNP applications in RNA vaccines.

### Ionizable lipids

Ionizable lipids (ILs), constituting 30–50% of LNP composition, play a pivotal role in RNA encapsulation and endosomal escape. They typically contain a hydrophilic ionizable cationic headgroup, a biodegradable linker, and one or more hydrophobic tails [57]. Their pKa (typically 6–7) is optimized to facilitate RNA binding in acidic environments while remaining neutral at physiological pH, minimizing toxicity [58]. When LNPs are internalized by cells via endocytosis, the acidic environment of the endosomes encourages fusion with the endosomal membrane, ensuring efficient release of the mRNA cargos into the cytoplasm.

**Figure 4:**
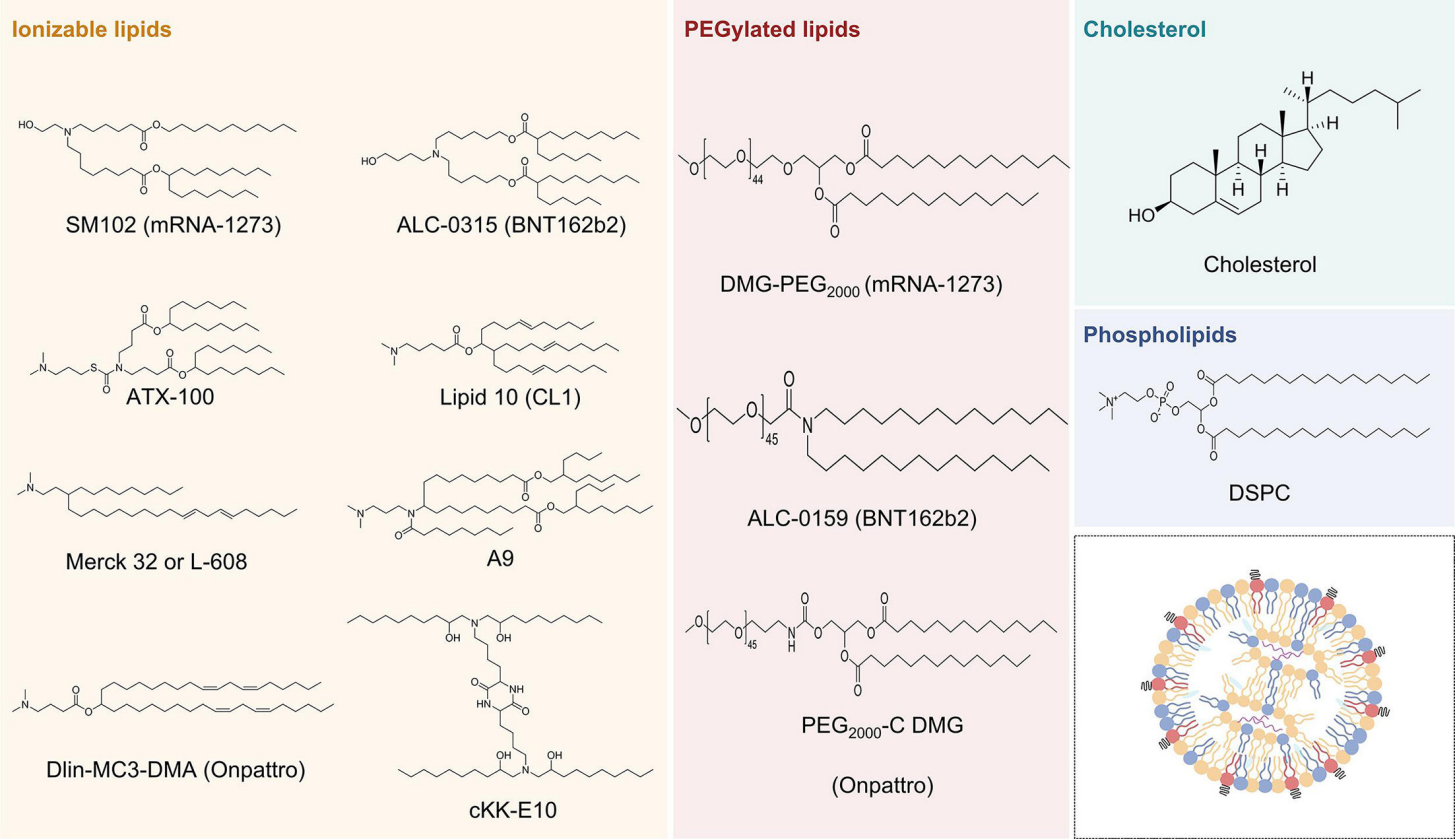
LNP components used in vaccines and vaccine candidates.

DODAP and DODMA are among the first ILs used for RNA delivery [59]. The first FDA-approved siRNA-LNP therapeutic, patisiran (Onpattro^®^), utilized Dlin-MC3-DMA ILs [60]. This breakthrough led to further IL innovations, including ALC-0315 (Pfizer/BioNTech BNT162b2) and SM-102 (Moderna mRNA-1273) [61]. ILs such as Dlin-MC3-DMA and L-608 (Merck 32) have been evaluated in respiratory syncytial virus (RSV) vaccine trials [62]. And ATX-100 was used in Arcturus' COVID-19 saRNA vaccine ARCT-154 [63]. In addition, some ILs remain undisclosed, such as those in the ARCoV vaccine co-developed by Suzhou Abogen, Walvax and the Academy of Military Medical Sciences of China (Figure 4) [64].

Recent IL advancements include polycyclic adamantane-based ILs (e.g. 11-AM) and cyclic imidazole-modified ILs (e.g. 93-O17S), which show potential for T cell targeting [65]. Furthermore, A18-Iso5-2DC18, with a cyclic amine headgroup, exhibits strong STING activation, promoting dendritic cell maturation [66]. Notably, an LNP incorporating 4N4T (four tertiary amine nitrogen atoms and four hydrophobic tails) lipids elicited stronger Th1-based immune responses and higher RBD-specific IgG and neutralizing antibody titers than SM-102-based vaccines [67].

### Cholesterol

Cholesterol, comprising 20–50% of LNPs, enhances membrane stability, delivery efficiency, and circulatory half-life [68]. Natural cholesterol is commonly used in current mRNA vaccines, but alternatives like β-sitosterol, a natural analogue, may further boost transfection efficiency by influencing cellular uptake [69]. However, the long-term safety of β-sitosterol and the costs associated with large-scale production still require further evaluation.

### Helper lipids

Helper lipids, the most commonly used are phospholipids, typically 10–20% of LNPs, forming a bilayer membrane that stabilizes RNA encapsulation and promotes efficient delivery. Saturated phosphatidylcholine lipids, such as 1,2-distearoyl-sn-glycero-3-phosphocholine, are widely used in RNA vaccines. In addition to their critical role in membrane formation, altering the polarity of phospholipid has been shown to influence the organ-targeting characteristics of LNPs [70].

### PEGylated lipids

PEGylated lipids make up a small fraction of the LNPs, typically around 1.5%. Despite their low abundance, increasing the content of PEGylated lipids improves the size distribution and overall stability of LNPs. This enhancement provides a protective shield against protein adsorption in the bloodstream, which in turn prevents recognition and clearance by the reticuloendothelial system and the mononuclear phagocyte system, thereby extending the circulation half-life of LNPs [68,71]. Research indicates that the length of the carbon chain in these PEGylated lipids affects their desorption rate. Presently, commercially available RNA vaccines use PEGylated lipids with 14-carbon chains, such as the ALC-0159 and DMG-PEG_2000_.

However, the widespread use of PEG in consumer products has led to pre-existing anti-PEG antibodies (APAs) in the population, which may accelerate LNP clearance (accelerated blood clearance, ABC), potentially reducing vaccine efficacy [72]. Repeated administration might further compromise vaccine effectiveness. Notably, between December 14 and 23, 2020, clinical data reported in the Vaccine Adverse Event Reporting System identified 21 cases of anaphylaxis in 1.89 million Pfizer/BioNTech vaccines. These reactions are most likely associated with sensitivity to PEG [73].

To address the challenges associated with PEG dilemma, various strategies have been explored, including enhancing mRNA expression efficiency to reduce dose requirements, replacing mRNA with saRNA that requires lower doses, and substituting PEGylated lipids with alternative materials. For example, Dong et al. demonstrated that replacing PEGylated lipids with pSar lipids improved mRNA delivery while maintaining safety [61]. Optimizing PEG terminal groups has also emerged as a promising strategy. Methoxy-PEGylation (MeO-PEG) is widely applied in numerous marketed nanomedicines, including MeO-PEG-DMG and ALC-0159 [74,75]. Zhan et al*.* discovered that hydroxyl-PEGylation (OH-PEG) at the PEG terminus effectively prevents binding by pre-existing APAs in humans [76]. Another innovative approach is substituting PEG with brush-shaped polymer lipids (BPLs), which exhibit lower APA affinity, prolonged circulation, and enhanced mRNA delivery. Xiao et al. developed high-density BPLs, demonstrating superior performance over conventional DMG-PEG2000 LNPs, particularly in repeated-dose studies [77]. These advancements provide new strategies to mitigate PEG-related limitations, paving the way for safer and more effective LNP-mRNA vaccines.

## Clinical applications and clinical trails

The recent achievements of mRNA vaccines, as seen in bnt162b2 (Pfizer-Biontech) and mRNA-1273 (Moderna), have sparked interest in their application to various infectious diseases like influenza, RSV, Zika virus, HIV, and more (Table 2). With their swift design, scalable production, and targeted immunogenicity, mRNA vaccines hold promise in addressing persistent challenges in vaccine development, presenting effective solutions for both emerging and re-emerging infectious diseases.

**Table 2:** Global clinical trials of RNA-based prophylactic vaccines for infectious diseases.

Pathogen	ClinicalTrials.Gov ID	Latest R&D status	Vaccine name	Sponsor	Main purpose
COVID-19	NCT05375838	Phase I/II	Elasomeran, mRNA-1073,mRNA-1010	ModernaTX, Inc.	Safety, reactogenicity, and immunogenicity of mRNA-1073 (SARS-CoV-2 and Influenza Vaccine). Compared with co-administered mRNA-1010 (Influenza) in healthy adults 18–75 years of age
	NCT06354998	Phase III	Elasomeran, mRNA-1273.815	ModernaTX, Inc.	Randomized, observer-blind, active-controlled of mRNA-1273.815 COVID-19 vaccine in previously vaccinated adults
	NCT05137236	Phase II	Elasomeran, mRNA-1283, mRNA-1283.211, mRNA-1283.529	ModernaTX, Inc.	Immunogenicity and safety of mRNA-1283 vaccine boosters for SARS-CoV-2
	NCT04927065	Phase II/III	Elasomeran, elasomeran + imelasomeran, mRNA-1273.213, mRNA-1273.815, mRNA-1273.231, mRNA-1273.211, mRNA-1273.529, elasomeran + davesomeran, mRNA-1273.617.2	ModernaTX, Inc.	Immunogenicity and safety of mRNA vaccine boosters for SARS-CoV-2 variants
	NCT04796896	Phase II/III	Elasomeran, elasomeran, imelasomeran	ModernaTX, Inc.	Safety, tolerability, reactogenicity, and effectiveness of mRNA-1273 SARS-CoV-2 vaccine in healthy children 6 months to less than 12 years of age
	NCT04649151	Phase II/III	Elasomeran, elasomeran + davesomeran	ModernaTX, Inc.	Safety, reactogenicity, and effectiveness of mRNA-1273 SARS-CoV-2 vaccine in healthy adolescents 12 to < 18 years of age
	NCT04380701	Phase I/II	BNT162a1, abdavomeran, tozinameran, pidacmeran	BioNTech SE	Safety and immunogenicity of four prophylactic SARS-CoV-2 RNA vaccines against COVID-19 using different dosing regimens in healthy and immunocompromised adults
	NCT05004181	Phase II	BNT162b2 (B.1.617.2), BNT162b2 (B.1.1.7), BNT162b2 (B.1.1.7 + B.1.617.2)	BioNTech SE	A phase II trial to evaluate the safety and immunogenicity of SARS-CoV-2 monovalent and multivalent RNA-based vaccines in healthy subjects
	NCT04649021	Phase II	Tozinameran	BioNTech SE	Safety and immunogenicity of SARS-CoV-2 mRNA vaccine (BNT162b2) in Chinese healthy population: A phase ii, randomized, placebo-controlled, observer-blinded study
	NCT04537949	Phase I/II	Ganulameran	BioNTech SE	Safety and immunogenicity of a prophylactic SARS-CoV-2 RNA vaccine (BNT162b3) against COVID-19 using different dosing regimens in healthy adults
	NCT04523571	Phase I	Abdavomeran	BioNTech SE	Safety and immunogenicity of SARS-CoV-2 mRNA vaccine (BNT162b1) in Chinese healthy subjects: A phase i, randomized, placebo-controlled, observer-blind study
	NCT04368728	Phase II/III	BNT162b2s01, abdavomeran, tozinameran	BioNTech SE, Pfizer	Safety, tolerability, immunogenicity, and efficacy of SARS-CoV-2 RNA vaccine candidates against COVID-19 in healthy individuals
	NCT04955626	Phase III	Riltozinameran, tozinameran + riltozinameran, tozinameran	BioNTech SE, Pfizer	Additional dose(s) of BNT162b2 in healthy individuals previously vaccinated with BNT162b2
	NCT06178991	Phase III	PF-07926307	BioNTech SE, Pfizer	Safety, tolerability, and immunogenicity of a combined modified RNA vaccine candidate against COVID-19 and influenza in healthy individuals
	NCT05472038	Phase II/III	Tozinameran + riltozinameran, tozinameran + famtozinameran,BNT162b5, tozinameran	BioNTech SE, Pfizer	Safety, tolerability, and immunogenicity of BNT162b RNA-based vaccine candidates in COVID-19 vaccine-experienced healthy individuals
	NCT06279871	Phase III	ARCT-2303	Arcturus Therapeutics, Inc.	Immunogenicity, reactogenicity, and safety of a saRNA COVID-19 vaccine (ARCT-2303), administered concomitantly with quadrivalent influenza vaccines, in adults
	NCT05037097	Phase I/II	ARCT-021, ARCT-165, ARCT-154	Arcturus Therapeutics, Inc.	Safety, reactogenicity, and immunogenicity of 3 SARS-CoV-2 RNA vaccine candidates in adults previously vaccinated and not previously vaccinated against SARS-CoV-2
	NCT05012943	Phase II/III	ARCT-154	Vinbiocare Biotechnology Joint Stock Company	Immunogenicity and efficacy of the SARS-CoV-2 saRNA vaccine ARCT-154 in adults
	NCT05517642	Phase III	Ad5-nCoV-IH, BNT162b2	CanSino Biologics Inc.	Immunogenicity, efficacy and safety of inhaled (IH) viral vectored vaccine (Convidecia, CanSino) as second booster dose against emerging variants of concern (VOC) of SARS-CoV-2 to prevent breakthrough infections
	NCT05373472	Phase II	CS-2034	CanSino Biologics Inc.	Safety and immunogenicity of COVID-19 mRNA vaccine in adults aged 18 years and older
	NCT05743335	Phase I	JCXH-221	Immorna Biotherapeutics, Inc.	Safety and immunogenicity of JCXH-221, an MRNA-based broadly protective COVID-19 vaccine
	NCT04863131	Phase I/II	EXG-5003	Fujita Health University	Safety and immunogenicity of intradermal SARS-CoV-2 vaccine EXG-5003 in healthy adults
	NCT05960097	Phase II	CV0701, CV0601	GlaxoSmithKline, CureVac	Safety, reactogenicity, and immunogenicity of a booster dose of investigational COVID-19 mRNA vaccines in healthy adults who previously received a complete primary vaccination series with or without booster dose(s)
	NCT05477186	Phase I	CV0501	GlaxoSmithKline	Safety and immunogenicity study of a booster dose of the investigational CV0501 mRNA COVID-19 vaccine in adults at least 18 years old
	NCT04758962	Phase I	GSK4184258A	GlaxoSmithKline	Safety, reactogenicity and immune response of a CoV-2 SAM (LNP) vaccine when administered intramuscularly on a 0, 1 month schedule in healthy adults 18 to 50 years of age
	NCT04652102	Phase II/III	CVnCoV	CureVac	Efficacy and safety of investigational SARS-CoV-2 mRNA vaccine CVnCoV in adults 18 years of age and older
	NCT05903118	Phase I/II	RBMRNA-176	Argorna Pharmaceuticals Co., LTD	Safety and immunogenicity of SARS-CoV-2 mRNA vaccine (RBMRNA-176) in healthy adults aged 18 years and older
	NCT05897190	Phase I	RBMRNA-405, CoronaVac^®^	Argorna Pharmaceuticals Co., LTD	Safety and immunogenicity of a SARS-CoV-2 mRNA vaccine (RBMRNA-405) as a booster in Chinese adults and older
	NCT05435027	Phase I	GRT-R912, GRT-R914, GRT-R918	Gritstone bio, Inc.	Safety and tolerability of GRT-R912, GRT-R914, and GRT-R918 administered as prime and/or boost in healthy adult participants and people living with HIV
	NCT05148962	Phase I	GRT-R910	Gritstone bio, Inc.	Safety, immunogenicity, and reactogenicity of a self-amplifying mRNA prophylactic vaccine boost against SARS-CoV-2 in previously vaccinated healthy adults 18 years and older
	NCT05329220	Phase III	ABNCoV2, tozinameran	Bavarian Nordic	Immunogenicity, safety, and tolerability of a single dose of ABNCoV2 vaccine in adult subjects previously vaccinated for SARS-CoV-2: A phase 3 trial in two parts-randomized, double-blind, active controlled and open-label, single-arm
	NCT05210179	Phase II	Tozinameran, Turkovac	Health Institutes of Turkey	Safety, efficacy, and immunogenicity of booster vaccination (TURKOVAC) against SARS-CoV-2
	NCT05668065	Phase III	CoronaVac, CoronaVac, BNT162b2	Institut Pasteur de Tunis	Immunogenicity of heterologous versus homologous prime boost schedule with mRNA and inactivated COVID-19 vaccines: A single-blinded, randomized, parallel group superiority trial
	NCT05352867	Phase II	LVRNA009	AIM Vaccine Co., Ltd.	Safety and immunogenicity of SARS-CoV-2 mRNA vaccine (LVRNA009) in Chinese people aged 18–59 years
	NCT05343871	Phase IV	Abdavomeran, ChAdOx1 nCoV-19, tozinameran	Albert B. Sabin Vaccine Institut	Immunogenicity and safety of full versus fractional dose of Pfizer/BioNTech, AstraZeneca, and Sinovac COVID-19 vaccines given as a booster dose at least 6 months after primary vaccination series or PCR-confirmed infection with SARS-CoV-2 in healthy adults
	NCT05188677	Phase III	SCB-2019, BNT162b2 Vaccine, Vaxzevria Vaccine, CoronaVac Vaccine	Clover Biopharmaceuticals AUS Pty Ltd	Immunogenicity and safety of adjuvanted recombinant SARS-CoV-2 trimeric S-protein subunit vaccine (SCB-2019), administered as a booster dose to adults, who previously received primary series of a COVID-19 vaccine
	NCT04775069	Phase IV	RBMRNA-405, CoronaVac^®^	Humanity & Health Medical Group Limited	Comparing the antibody response of subjects with chronic liver disease to mRNA, inactivated virus and adenovirus vector COVID-19 vaccines
	NCT05030974	Phase IV	Elasomeran, JNJ-78436735	University Medical Center Groningen	Randomized multicenter study by the REnal patients COVID-19 VACcination (RECOVAC) Consortium
	NCT04566276	Phase I/II	ChulaCov19	Chulalongkorn University	Safety, tolerability, and immunogenicity of the ChulaCov19 vaccine in healthy adults
	NCT04569383	Phase I	MVA-SARS-2-S, tozinameran	Universitätsklinikum Hamburg-Eppendorf	Safety, tolerability and immunogenicity of two ascending doses of the candidate vaccine MVA-SARS-2-S and heterologous booster vaccinations with a licensed vaccine against COVID-19
	NCT04765436	Phase I	PTX-COVID19-B	Providence Therapeutics Holdings Inc.	Safety, tolerability, and immunogenicity of PTX-COVID19-B vaccine in healthy seronegative adults aged 18–64
	NCT04821674	Phase I/II	DS-5670	Daiichi Sankyo Co., Ltd.	Safety, immunogenicity and recommended dose of DS-5670a (COVID-19 vaccine) in Japanese healthy adults and elderly subjects
	NCT05305573	Phase II	Bimervax, tozinameran	Hipra Scientific, S.L.U	Immunogenicity and safety of a booster vaccination with a recombinant protein RBD fusion dimer candidate (PHH-1V) against SARS-CoV-2, in adults fully vaccinated with adenovirus vaccine against COVID-19
	NCT05197153	Phase II	Elasomeran, ChAdOx1 nCoV-19	Medigen Vaccine Biologics Corp.	Safety, tolerability, and immunogenicity of heterologous booster dose with AZD1222, mRNA-1273, or MVC-COV1901 COVID-19 vaccine in adults
	NCT05175742	Phase II	PTX-COVID19-B, tozinameran	Providence Therapeutics Holdings Inc.	Safety, tolerability, and immunogenicity of PTX-COVID19-B compared to Pfizer-BioNTech COVID-19 vaccine in healthy seronegative adults aged 18 to 64 years
	NCT05144139	Phase I/II	SW0123	Stemirna Therapeutics	Safety, immunogenicity and immune persistence of COVID-19 mRNA vaccine in healthy people aged 18 years and above
	NCT05049226	Phase II	ChAdOx1 nCoV-19, tozinameran	Mahidol University	Immunogenicity and safety of third dose vaccination with AstraZeneca COVID-19 vaccine or Pfizer/BioNTech COVID-19 vaccine among Thai Adults receiving two doses of Sinovac
COVID-19, influenza	nct06097273	Phase III	mRNA-1083	ModernaTX, Inc.	Safety, reactogenicity, and immunogenicity of mRNA-1083 (SARS-CoV-2 and influenza) vaccine in healthy adult participants, ≥50 years of age
	NCT05596734	Phase I/II	PF-07252220, PF-07926307, tozinameran + famtozinameran	BioNTech SE, Pfizer	Safety, tolerability, and immunogenicity of combined modified RNA vaccine candidates against COVID-19 and influenza in healthy individuals
	NCT04969276	Phase II	Elasomeran, Fluzone High-Dose Quadrivalent	Sanofi Pasteur, a Sanofi Company	Safety and immunogenicity of Fluzone^®^ high-dose quadrivalent (influenza vaccine), 2021–2022 formulation and a third dose of Moderna COVID-19 vaccine (mRNA-1273 vaccine) administered either concomitantly or singly in adults 65 years of age and older previously vaccinated with a 2-dose schedule of Moderna COVID-19 vaccine
	NCT05970887	Phase IV	Tozinameran + famtozinameran	Catholic Kwandong University	Immunogenicity and safety of concomitant administration of Omicron-containing bivalent COVID-19 vaccines with seasonal influenza vaccines
COVID-19, influenza, RSV	NCT05585632	Phase I	mRNA-1045, elasomeran, elasomeran + imelasomeran, mRNA-1010, mRNA-1230, mRNA-1345	ModernaTX, Inc.	Safety, reactogenicity, and immunogenicity of multi-component vaccines mRNA-1045 (influenza and RSV) or mRNA-1230 (influenza, RSV, and SARS-CoV-2) compared with mRNA-1010 (influenza), mRNA-1345 (RSV), and mRNA-1273.214 (SARS-CoV-2) vaccines in healthy adults 50–75 years of age
	NCT05330975	Phase III	Elasomeran, elasomeran + imelasomeran, Afluria Quadrivalent, Afluria (trivalent), mRNA-1345	ModernaTX, Inc.	Safety, tolerability, and immunogenicity of mRNA-1345, an mRNA vaccine targeting respiratory syncytial virus (RSV), when given alone or coadministered with a seasonal influenza vaccine or SARS-CoV-2 vaccine and when given as an open-label boost at 1 year following a primary dose in adults ≥ 50 years of age
Covid-19, RSV	NCT05886777	Phase II	PF-06928316, tozinameran + famtozinameran	Pfizer	Safety, tolerability, and immunogenicity of combined vaccine candidate(s) against infectious respiratory illnesses, including COVID-19 and RSV, in healthy individuals
Covid-19, HPV	NCT05119855	Phase III	Elasomeran, Gardasil 9	Merck Sharp & Dohme LLC	Safety and immunogenicity of 2-dose regimens of 9vhpv and mRNA-1273 SARS-CoV-2 vaccines where the first dose of each vaccine are given concomitantly in boys and girls 9 to 11 Years of age
COVID-19, S. pneumoniae	NCT05158140	Phase III	mRNA-1273, V110, V114	Merck Sharp & Dohme LLC	Safety, tolerability, and immunogenicity of the concomitant administration of either 23-valent pneumococcal polysaccharide vaccine or 15-valent pneumococcal conjugate vaccine with a booster dose of SARS-CoV-2 mRNA vaccine in healthy adults 50 years of age or older.
	NCT04887948	Phase III	20vPnC	Pfizer	Safety and immunogenicity of 20 valent pneumococcal conjugate vaccine when coadministered with a booster dose of BNT162b2 in adults 65 Years of age and older
Influenza	NCT05540522	Phase III	PF-07252220	Pfizer	Efficacy, safety, tolerability, and immunogenicity of a modified RNA vaccine against influenza compared to licensed inactivated influenza vaccine in healthy adults 18 years of age or older
	NCT05227001	Phase I	PF-07836391, PF-07836395, PF-07836396, PF-07836394, PF-07852352, PF-07867246	Pfizer	Safety, tolerability, and immunogenicity of saRNA vaccine preparations against influenza in healthy individuals
	NCT03076385	Phase I	VAL-506440	ModernaTX, Inc.	Safety and immunogenicity of H10N8 antigen mRNA in healthy adult subjects
	NCT05827978	Phase III	mRNA-1010, Fluarix Quadrivalent (FLU D-QIV)	ModernaTX, Inc.	Immunogenicity, reactogenicity and safety of mRNA-1010 seasonal influenza vaccine in adults 18 years and older
	NCT05333289	Phase I/II	mRNA-1030, mRNA-1010, mRNA-1020	ModernaTX, Inc.	Safety, reactogenicity, and immunogenicity of mRNA-1020 and mRNA-1030 candidate seasonal influenza vaccines in healthy adults
	NCT03345043	Phase I	VAL-339851	ModernaTX, Inc.	Safety and immunogenicity of VAL-339851 in healthy subjects
	NCT05566639	Phase III	mRNA-1010	ModernaTX, Inc.	Safety and efficacy of mRNA-1010 candidate seasonal influenza vaccine in adults 50 years and older
Influenza	NCT05566639	Phase III	mRNA-1010	ModernaTX, Inc.	Safety and efficacy of mRNA-1010 candidate seasonal influenza vaccine in adults 50 years and older
	NCT05972174	Phase I/II	mRNA-1018	ModernaTX, Inc.	Safety, reactogenicity, and immunogenicity of mRNA-1018 pandemic influenza candidate vaccines in healthy adults ≥ 18 years of age
	NCT05827068	Phase I/II	mRNA-1012.1, mRNA-1010.3, mRNA-1011.2, mRNA-1010, mRNA-1011.1, mRNA-1010.2	ModernaTX, Inc.	Safety, reactogenicity, and immunogenicity of mRNA-1011.1, mRNA-1011.2, and mRNA-1012.1 candidate seasonal influenza vaccines in healthy adults 50 to 75 years of age
	NCT05868382	Phase II	mRNA-1010.4, mRNA-1010, mRNA-1010.6	ModernaTX, Inc.	Safety, reactogenicity, and immunogenicity of mrna vaccine candidate variations in healthy adults 18 to 49 years of age
	NCT06028347	Phase I	SQ012	Seqirus	Safety, reactogenicity and immunogenicity of an investigational self-amplifying MRNA influenza vaccine in healthy adults
	NCT05829356	Phase I	H3 mRNA/LNP Vaccine (sanofi), Flublok Quadrivalent	Sanofi Pasteur, a Sanofi Company	Early safety data reviews to assess safety and immunogenicity of one monovalent modified influenza mRNA vaccine encapsulated in LNP, in adults aged 18 to 49 years and 60 years and above.
	NCT05650554	Phase I/II	Fluzone Quadrivalent, MRT5413, Flublok Quadrivalent, Fluzone High-Dose Quadrivalent	Sanofi Pasteur, a Sanofi Company	Safety and immunogenicity of quadrivalent influenza mRNA vaccine MRT5413 in adults aged 18 Years and older
	NCT05553301	Phase I/II	MRT5407, Fluzone Quadrivalent, Flublok Quadrivalent, Fluzone High-Dose Quadrivalent	Sanofi Pasteur, a Sanofi Company	Safety and immunogenicity of quadrivalent influenza mRNA vaccine MRT5407 in adults aged 18 Years and older
	NCT05624606	Phase I/II	Fluzone Quadrivalent, MRT5410, Flublok Quadrivalent, Fluzone High-Dose Quadrivalent	Sanofi Pasteur, a Sanofi Company	Safety and immunogenicity of quadrivalent influenza mRNA vaccine MRT5410 in adults aged 18 years and older
	NCT05426174	Phase I	SP0273, Fluzone High-Dose Quadrivalent	Sanofi Pasteur, a Sanofi Company	Safety and immunogenicity of monovalent mRNA vaccine in adult participants 18 years of age and older
	NCT03814720	Phase I	VRC-FLUNPF099-00-VP	NIAID	Dose, safety, tolerability, and immunogenicity of an influenza H1 stabilized stem ferritin vaccine, VRCFLUNPF099-00-VP, in healthy adults
	NCT05446740	Phase I	FLU SV mRNA	GlaxoSmithKline, CureVac	Safety, reactogenicity and immunogenicity of an mRNA-based monovalent influenza vaccine candidate in healthy younger and older adults
	NCT05252338	Phase I	CVSQIV	GlaxoSmithKline, CureVac	Safety, reactogenicity and immunogenicity of the investigational seasonal quadrivalent influenza mRNA vaccine CVSQIV administered intramuscularly in healthy younger and older adults
Influenza, RSV	NCT05788237	Phase I	PF-07252220, PF-06928316	Pfizer	Safety, tolerability, and immunogenicity of respiratory combination vaccine candidates in older adults
	NCT06060457	Phase III	Fluzone High-Dose Quadrivalent, mRNA-1345	ModernaTX, Inc.	Safety, tolerability, and immunogenicity of mRNA-1345, an mRNA vaccine targeting respiratory syncytial virus, when coadministered with a high-dose, quadrivalent seasonal influenza vaccine in adults ≥ 65 years of age
HPIV, hMPV	NCT04144348	Phase I	mRNA-1653	ModernaTX, Inc.	Safety and immunogenicity of mRNA-1653, a combined human metapneumovirus (hMPV) and parainfluenza virus type 3 (PIV3) vaccine when administered to adults, and to children 12 to 59 months of age with serologic evidence of prior exposure
	NCT03392389	Phase I	mRNA-1653	ModernaTX, Inc.	Safety, reactogenicity, and immunogenicity of mRNA-1653, a combined human metapneumovirus and human parainfluenza virus type 3 vaccine, when administered to healthy adults
ZIKV	NCT04917861	Phase II	mRNA-1893	ModernaTX, Inc.	Safety, tolerability, and immunogenicity of Zika vaccine mRNA-1893 in adults aged 18 through 65 years and living in endemic and non-endemic flavivirus areas
	NCT03014089	Phase I	mRNA-1325	ModernaTX, Inc.	Safety and immunogenicity of mRNA 1325 zika vaccine in healthy adults in a non-endemic Zika region
	NCT04064905	Phase I	mRNA-1893	ModernaTX, Inc.	Safety, tolerability, and immunogenicity of Zika vaccine mRNA-1893 in healthy flavivirus seropositive and seronegative adults
HIV-1	NCT03547245	Phase I	mRNA-1644	International AIDS Vaccine Initiative	Safety and immunogenicity of eOD-GT8 60mer vaccine, adjuvanted in HIV-uninfected, healthy adult volunteers
RABV	NCT04062669	Phase I	GSK3903133A	GlaxoSmithKline	Safety and immunogenicity of GSK's rabies G SAM (CNE) vaccine [GSK3903133A] in healthy adults
	NCT03713086	Phase I	CV7202	CureVac	Safety, reactogenicity and immunogenicity of one or two administrations of candidate rabies mRNA vaccine CV7202 in healthy adult subjects
	NCT02241135	Phase I	Nadorameran	CureVac	Safety and immunogenicity trial of an investigational RNActive® Rabies vaccine (CV7201) in healthy adults
CMV	NCT04232280	Phase II	mRNA-1647	ModernaTX, Inc.	Safety and immunogenicity of cytomegalovirus vaccine mRNA-1647 in healthy adults
	NCT03382405	Phase I	mRNA-1443, mRNA-1647	ModernaTX, Inc.	Safety, reactogenicity, and immunogenicity of cytomegalovirus vaccines mRNA-1647 and mRNA-1443 when administered to healthy adults
VZV	NCT05871541	Phase I	JCXH-105	Immorna Biotherapeutics, Inc.	Safety and immunogenicity of a Herpes Zoster (HZ) v accine, JCXH-105, in healthy subjects 50 to 69 years of age.
NiV	NCT05398796	Phase I	mRNA-1215	NIAID	Safety, tolerability and immunogenicity of a nipah virus (NiV) mRNA vaccine, mRNA-1215, in healthy adults
PfCSP	NCT05581641	Phase I	BNT165b1	BioNTech SE	Safety, tolerability and immunogenicity of an investigational RNA-based vaccine for active immunization against malaria
CHIKV	NCT03325075	Phase I	mRNA-1388	ModernaTX, Inc.	Safety and immunogenicity of mRNA-1388 in healthy adults in a non-endemic Chikungunya region

The data in Table 2 were obtained from ClinicalTrials.gov.

### SARS-CoV-2

mRNA-based COVID-19 vaccines represent the most extensively explored mRNA vaccine platform to date. Comirnaty^®^ (BNT162b2) was the first mRNA vaccine authorized for emergency use by the FDA on December 11, 2020, and by the EMA on December 21, 2020 [78]. Shortly thereafter, Moderna’s mRNA-1273 vaccine also received approval. Both vaccines encode the full-length spike protein of SARS-CoV-2 and demonstrated 95.0% and 94.1% efficacy, respectively, in Phase III clinical trials [79]. To combat viral mutations, new vaccine formulations continue to be developed. For example, CSPC Pharmaceutical Group Ltd. developed SYS6006, an mRNA-based vaccine encoding the spike protein sequence with key mutations from Delta, Omicron BA.4, BA.5, and BF.7 strains, which received emergency use authorization (EUA) in China [80–82]. Moderna’s mRNA-1283, which encodes only the RBD and N-terminal domain of the spike protein, exhibited enhanced immune responses against the Omicron variant BA.4/BA.5 and the original virus strains compared to mRNA -1273.222 in Phase III clinical trials [83]. Additionally, mRNA-1283 exhibits improved stability at standard refrigeration temperatures (2–8℃), simplifying storage and distribution [84].

Combination vaccines represent a promising next-generation strategy. Moderna’s mRNA-1083, which integrates the influenza vaccine mRNA-1010 with the COVID-19 vaccine mRNA-1283, is currently in Phase III trials after demonstrating comparable or superior efficacy to licensed quadrivalent influenza vaccines and mRNA-1273 in earlier trials [85,86]. Beyond conventional mRNA vaccines, saRNA vaccines are under investigation. Arcturus Therapeutics' ARCT-154, an saRNA-based COVID-19 vaccine, demonstrated broader efficacy, longer-lasting immune protection, and reduced antigen dose requirements compared with BNT162b2, marking the first published clinical evidence of saRNA vaccine efficacy [87,88].

Clinical trials have also assessed the safety and immunogenicity of COVID-19 mRNA vaccines in special populations. Studies indicate that these vaccines elicited strong immune responses with acceptable tolerability in both elderly and pediatric populations [89–91]. Moreover, a six-month follow-up study of individuals aged 12 and older who received two doses of BNT162b2 reported an efficacy rate exceeding 86% and a favorable long-term safety profile [92]. These findings provide crucial support for broad vaccine deployment in diverse age groups.

### Influenza

The segmented RNA genome of influenza viruses allows for the emergence of multiple subtypes and frequent antigenic drift, posing challenges for vaccine development. Currently, at least 18 distinct influenza A virus subtypes are circulating globally [93]. Multivalent vaccines encoding antigens for multiple subtypes offer a promising solution, which is difficult with conventional vaccines technology but feasible with RNA-based vaccine platforms.

Moderna’s mRNA-1010, a quadrivalent seasonal influenza vaccine encoding the full-length hemagglutinins (HA) of four influenza strains (A/H1N1, A/H3N2, B/Victoria, and B/Yamagata). In Phase I/II clinical trials (NCT04956575), single doses of mRNA-1010 induced hemagglutination inhibition (HAI) titers higher than those from standard inactivated vaccines against influenza A strains, while producing comparable HAI titers for influenza B strains [94,95]. However, the results of Phase III clinical trial were less favorable. mRNA-1010 was associated with higher adverse event rates compared with traditional vaccines, similar immunogenicity against influenza A, and inferior immunogenicity against influenza B [96]. These findings highlight the need for further optimization of mRNA vaccine platforms or antigen design to enhance efficacy and safety. Beyond conventional mRNA vaccines, Pfizer has developed saRNA-based influenza vaccine candidates. A Phase I clinical trial has been completed, though results have not yet been disclosed (NCT05227001, data from https://clinicaltrials.gov).

### Respiratory syncytial virus

RSV is a single-stranded, negative-sense RNA virus and the leading cause of bronchitis and pneumonia in infants, young children, the elderly, and immunocompromised individuals. On May 31, 2024, the FDA approved Moderna’s mRNA-1345, encoding a stabilized prefusion F glycoprotein, marking the first RSV mRNA vaccine for older adults (≥60 years) [97], Sanofi’ SP0256, an RSV mRNA vaccine for older adults, showed promising Phase I/II clinical trials results and is currently in a Phase IIb trial in combination with human metapneumovirus (hMPV) (NCT06134648, NCT06237296). Sanofi also plans to develop a trivalent mRNA vaccine targeting RSV, hMPV, and parainfluenza virus [98].

While RSV mRNA vaccines have demonstrated preventive efficacy, safety concerns remain. The most common adverse reactions (ARs) include injection site pain, fatigue and myalgia (systemic), which are generally mild to moderate and transient [99]. However, in July 2024, the FDA placed an emergency hold on the Phase I clinical trial of mRNA-1365-P101 due to a potential safety signal [100]. Specifically, an imbalance in severe lower respiratory tract infections caused by RSV was observed, with a higher incidence in the vaccine group than in controls. The underlying cause remains unclear, highlighting the need for continued safety evaluations as mRNA vaccines become more widely used. To date, no broad-spectrum universal vaccine has been successfully developed for all age groups.

### HIV

Developing an HIV vaccine has posed a longstanding challenge. Despite decades of research, numerous HIV vaccine candidates have failed in Phase III trials, and no vaccine has been approved for human use [101]. Moderna is currently conducting Phase I clinical studies to evaluate the safety and immunogenicity of the eOD-GT8 60mer mRNA vaccine (mRNA-1644) and the Core-g28v2 60mer mRNA vaccine (mRNA-1644v2-Core) in HIV-1 uninfected adults (NCT05001373). This trial aims to assess the ability of mRNA-based vaccines to elicit specific B-cell responses and drive their early maturation toward generating broadly neutralizing antibodies (bnAbs) [102]. Additionally, Moderna has launched another Phase I trial for the mRNA-1574 vaccine candidate, which is designed to encode native-like HIV trimers. The main endpoints of this trial are to evaluate the safety profile and immunogenicity of this experimental vaccine, with a specific focus on immune responses targeting HIV trimers (NCT05217641) [103]. As of now, results from the above trials have not been disclosed.

### Other pathogens

mRNA vaccines are also being explored for various other viral infections. Moderna developed mRNA-1325 (NCT03014089) and mRNA-1893 (NCT04064905), both encoding the Zika premembrane (prM) and envelope (E) proteins. Phase I clinical trials demonstrated that mRNA-1893 elicited stronger serum-neutralizing antibody responses and better tolerability than mRNA-1325 after two doses [104,105]. For cytomegalovirus (CMV), Moderna’s Mrna-1647, comprising six mRNA sequences encoding the CMV pentamer complex and glycoprotein B (gB) antigens, demonstrated potent and durable humoral responses in Phase I/II trials, with superior neutralizing activity and antibody-dependent cellular cytotoxicity compared to the traditional gB/MF59 vaccine [106,107]. Currently, this vaccine is undergoing Phase III trials. In malaria research, BioNTech’s BNT165b1, an mRNA vaccine targeting Plasmodium falciparum circumsporozoite protein (PfCSP), entered Phase I clinical trials in 2022 (NCT05581641), representing a step toward effective malaria vaccines [108].

### Terminated projects

Despite significant progress, several mRNA vaccine projects have faced challenges, underscoring the need for continuous optimization in vaccine design. For example, the early Zika virus mRNA vaccine (mRNA-1325) revealed that single amino acid differences disrupted virus-like particle formation, reducing immunogenicity and limiting efficacy [105]. Similarly, CureVac’s COVID-19 vaccine (CVnCoV) exhibited only 47% efficacy in Phase III trials, largely due to its lack of nucleoside modifications, which heightened innate immune activation and diminished potency [109]. In contrast, vaccines from Pfizer-BioNTech and Moderna, incorporating modified nucleosides, demonstrated significantly higher efficacy. Additionally, CureVac’s rabies vaccine (CV7201), which used protamine as a delivery system, showed route-dependent efficacy and caused adverse reactions in 78% of Phase I participants [110]. Development shifted to CV7202, which employed LNPs for improved delivery efficiency and reduced reactogenicity [111]. As viral variants continue to emerge, single-epitope vaccines may lack broad-spectrum protection. Future designs should consider multivalent strategies and optimize mRNA sequences, delivery systems, and adaptability to evolving pathogens to enhance vaccine efficacy and safety.

## Challenges of mRNA vaccines

### Stability and storage

mRNA-LNP vaccines, such as mRNA-1273 and BNT162b2, require frozen storage at −20℃ and −60℃ to −80℃, respectively, posing logistical challenges in resource-limited regions [112]. Lyophilization (freeze-drying) offers a promising solution by enhancing stability and shelf-life, potentially enabling ambient temperature storage [113]. However, the process must preserve key characteristics, such as particle size, polydispersity index, encapsulation efficiency, mRNA integrity, and immunogenicity [114]. Recent studies have demonstrated the feasibility of lyophilizing mRNA-LNP formulations. For example, Muramatsu *et al.* showed that mRNA-LNPs formulated in 5 mM Tris buffer (pH 8) with 10% sucrose and 10% maltose maintained their stability at room temperature for 12 weeks and at 4℃ for 24 weeks [115]. Similarly, Ai et al. described a lyophilized Omicron mRNA vaccine that remained stable at 25℃ for over six months, retaining robust humoral and cellular immune responses [116]. Moderna’s CMV vaccine (mRNA-1647) is undergoing Phase III clinical trials in healthy participants aged 16 to 40 years (NCT05085366) [117]. This lyophilized formulation has demonstrated stability at 5℃ for up to 18 months. Beyond lyophilization, alternative drying techniques, such as spray drying, spray freeze drying, thin-film freeze-drying, and supercritical spray drying, are being explored to optimize stability and vaccine performance [118]. Further research is needed to refine these approaches for large-scale implementation.

### Extrahepatic tissues delivery

LNP-mRNA systems exhibit an inherent liver affinity, mainly due to ApoE-mediated uptake by hepatocytes, especially Kupffer cells and hepatic sinusoidal endothelial cells, making targeted delivery to extrahepatic tissues a significant challenge [119,120].

Several strategies are being explored to enhance selective organ targeting (SORT) [121]. Lipid modifications, LNPs synthesized with 5A2-SC8 lipids, combined with the addition of the cationic lipid DOTAP, can reduce ApoE binding and enhance targeting to other tissues, such as the lungs and spleen [122]. Adjusting the surface charge of LNPs is another effective approach to reduce liver uptake [123]. Moreover, surface modification of LNPs, such as coupling targeted molecules (like antibodies, peptides, or polysaccharides) to the LNP surface, represents another promising method for improving extrahepatic tissues delivery efficiency [124]. The choice of administration route also plays a crucial role in delivery efficiency, with nasal and intradermal administration enabling direct access to certain tissues. Furthermore, studies indicate that LNP size influences biodistribution, with nanoparticles in the 20–200 nm range demonstrating enhanced uptake by dendritic cells (DCs), thereby improving immune responses [120].

While extrahepatic mRNA delivery remains an area of active research, substantial progress has been made in targeting the lungs, tumors, and central nervous system through lipid chemistry innovations and ligand-based modifications. Future advancements must focus on refining targeting accuracy, ensuring safety, and optimizing large-scale production to expand the clinical applicability of mRNA vaccines.

## Conclusion

LNP-mRNA vaccines represent a distinct advancement over traditional vaccines, with the successful application of BNT162b2 and mRNA-1273 during COVID-19 pandemic, highlighting the unprecedented adaptability and efficacy of mRNA vaccine technology in addressing infectious diseases. A comprehensive understanding of mRNA vaccines composition and optimization strategies is essential for further advancing this transformative technology. Optimizing RNA stability and translation efficiency has been a key focus, involving modifications such as alternative RNA types, nucleotide chemical alterations, codon optimization, and refined non-coding regions. Simultaneously, advancements in LNP formulations have enhanced cellular uptake, reduced off-target effects, and improved biodistribution. However, several challenges remain that must be addressed to realize the full potential of mRNA vaccines.

The intrinsic instability of mRNA necessitates a strict cold chain, limiting accessibility in resource-limited regions where ultra-low temperature storage is often impractical. The development of lyophilized mRNA-LNPs, which enable storage at 4℃ for up to six months, represents a major breakthrough. However, the impact of lyophilization buffer composition, processing conditions, and storage parameters on LNP integrity requires further investigation. Another major challenge is LNP liver accumulation, largely due to ApoE-mediated uptake. To overcome this, extensive research has focused on organ-specific LNP technologies, including high-throughput lipid screening, formulation optimization, and antibody-modified delivery systems. A particularly promising approach is SORT technology, which incorporates charged lipids (SORT lipids) into conventional LNP formulations, enabling targeted mRNA delivery to extrahepatic tissues.

Beyond technological challenges, ensuring equitable access to mRNA vaccines remains a critical global priority. The WHO’s fair allocation mechanism for COVID-19 vaccines through COVAX highlights the need to reduce distribution disparities and ensure mRNA vaccines reach low- and middle-income countries (LMICs). To achieve this, optimizing cold chain logistics, improving vaccine thermostability, and reducing production costs are essential. Strengthening local mRNA manufacturing capabilities in LMICs could also enhance regional vaccine security and pandemic preparedness, as evidenced by WHO-led initiatives promoting technology transfer hubs for mRNA vaccine production.

Furthermore, concerns regarding immunogenicity and long-term safety demand continued investigation. While recent clinical trials have shown promising immune responses and safety profiles, variations in vaccine efficacy across different populations emphasize the need for further validation. Additionally, the precise *in vivo* immune mechanisms of mRNA vaccines remain incompletely understood, necessitating deeper exploration to optimize immune response durability and broad applicability across diverse LNP formulations.

Addressing these scientific, logistical, and ethical challenges is crucial for expanding mRNA vaccine accessibility worldwide. Continued technological innovation, coupled with global cooperation in vaccine allocation and infrastructure development, will be instrumental in realizing the full potential of mRNA vaccines in combating infectious diseases on a global scale.

**Summary**

- mRNA vaccines possess distinct advantages, including rapid development timelines, scalable production, and the ability to encode multiple antigens, showcasing their significant potential as next-generation vaccines.
- Lipid nanoparticles remain the leading delivery system for mRNA vaccines. Advances in lipid design, formulation optimization, and selective organ targeting strategies continue to enhance their safety, transfection efficiency, and biodistribution.
- Modifications to mRNA structure, including nucleoside modifications, codon optimization, and adjustments to untranslated regions, significantly improve stability, translation efficiency, and immunogenicity, thereby enhancing vaccine efficacy.
- Ongoing clinical trials for various infectious diseases, such as SARS-Cov-2, respiratory syncytial virus, influenza, Zika virus, and HIV, have yield promising immunogenicity and safe data, with several candidates advancing to next-stage development.
- Despite significant progress, key challenges remain, including long-term safety assessment, potential immunogenicity management, cold-chain requirements, and extrahepatic delivery improvements, all of which are critical for the advancement of future mRNA vaccine formulations.

## Abbreviations

IVT: *in vitro* transcription
LNPs: lipid nanoparticles
SORT: selective organ targeting
TLRs: Toll-like receptors
sa-RNA: self-amplifying RNA
